# Web-based data collection: detailed methods of a questionnaire and data gathering tool

**DOI:** 10.1186/1742-5573-3-1

**Published:** 2006-01-04

**Authors:** Charles J Cooper, Sharon P Cooper, Deborah J del Junco, Eva M Shipp, Ryan Whitworth, Sara R Cooper

**Affiliations:** 1 Department of Epidemiology and Biostatistics, Texas A&M School of Rural Public Health, College Station, USA; 2 Centers for Health Promotion and Prevention Research, The University of Texas School of Public Health, Houston, USA; 3 Department of Psychology, The University of Illinois, Champaign, IL

## Abstract

There have been dramatic advances in the development of web-based data collection instruments. This paper outlines a systematic web-based approach to facilitate this process through locally developed code and to describe the results of using this process after two years of data collection. We provide a detailed example of a web-based method that we developed for a study in Starr County, Texas, assessing high school students' work and health status. This web-based application includes data instrument design, data entry and management, and data tables needed to store the results that attempt to maximize the advantages of this data collection method. The software also efficiently produces a coding manual, web-based statistical summary and crosstab reports, as well as input templates for use by statistical packages.

Overall, web-based data entry using a dynamic approach proved to be a very efficient and effective data collection system. This data collection method expedited data processing and analysis and eliminated the need for cumbersome and expensive transfer and tracking of forms, data entry, and verification. The code has been made available for non-profit use only to the public health research community as a free download [1].

## Background

### Introduction

Since the introduction of computers, there has been an evolution of improvements in data collection methods corresponding to advances in technology. Specifically, there have been dramatic advances in the development of web-based data collection instruments. The purpose of this paper is to outline a systematic web-based approach to facilitate this process through locally developed code and to describe the results of using this process after two years of data collection. We present a summary of the evolution of computerized data collection methods including web development methods. We provide a detailed example of a web-based method that we developed for a study in Starr County, Texas, assessing high school students' work and health status. The example presented in this paper includes questionnaire design, data entry, and data management that attempt to maximize the advantages of this data collection method.

We use the term "dynamic" to refer to the process of generating a web-based questionnaire from a database of questions, possible responses, and controls at the time of connection by the client browser using an application running on the web server. This method is in contrast to the static method of pre-designing the questionnaire as an HTML web form and placing it on the server so that it can be loaded and displayed through the client browser at the time of connection. Details of this dynamic method and its advantages are reviewed and the code is available for non-profit use only to the public health research community as a free download. A link to the current version of our software can be found in this reference [1].

### Evolution of computerized data collection methods

In the early 1970s, large multi-center trials such as the Hypertension Detection and Follow-up Program collected massive amounts of data through questionnaires completed at physicians' offices via paper forms and transferred these forms to a central site where the data were manually entered into mainframe computers [2,3]. In the early 1980s, studies such as the Systolic Hypertension in the Elderly Program [4,5] improved on the quality and faster availability of the data by using personal computers at the local sites for data entry and then for transfer of data electronically via modems to the mainframes. In the middle and late 1990s, other techniques began to emerge that used the internet for data collection, such as direct mailings of questionnaires through the internet and web-based data entry and management systems [6-9]. More recently, researchers are using newer techniques of remote entry such as using Wireless Markup Language using handheld devices like mobile phones [10] and systems that use computer-assisted personal and telephone interviewing [11].

### Development of web-based data collection

One of the most significant advancements in remote entry is the process of entering data on a form accessed on the Web. This method has become a popular way to collect data because access to the internet has expanded dramatically, allowing data to be entered directly into a central database. It also provides less dependency on specific types of equipment for entering data. Web-based methods allow for instant editing checks as responses are entered, and, if desired, allows for many of the traditional techniques for inputting responses such as textboxes, dropdowns, checkboxes or other styles that are available through web programming without additional software installed on the client other than a web browser.

Early web developers had to use Hyper Text Markup Language (HTML) to develop web forms that were displayed through client browsers and allowed data to be entered and submitted to a server. The JavaScript programming language was used to allow data to be validated before it was submitted. Enhancements to HTML through Dynamic HTML (DHTML) and Cascading Style Sheets (CSS) [12] gave developers the ability to better control the appearance of the web forms and graphical images. Server side languages (such as Java, Active Server Pages (ASP), and Personal Home Page (PHP)) are used to produce applications on the server that execute at the time of connection to the web site. These applications produce and render questionnaires based on information contained in databases, files or other sources maintained on the server. These languages are also used to build server applications that more extensively examine the data for errors when it is submitted by the remote computer. More recently, the eXtensible Markup Language (XML) has been adapted by many developers as a new tool for transmitting data systematically. When used in combination with eXtensible Stylesheet Language (XSL), richer and more powerful systems have been developed that make use of the built in validation techniques and the use of controls and images for collecting and displaying data [13,14]. In October 2003, the internet standards W3C committee submitted new recommendations for an Xform standard that uses XML as the base for producing the next generation of data collection instruments for the web [15]. With the emergence of the ASP.Net programming language [16], and enhancements to JAVA, PHP and other programming languages, developers now have a vast array of tools in which applications can be developed. An extensive review of many of these techniques and related references can be found in Vasu et al. [11], and Gunn [17].

Web-based data collection also has its disadvantages. Researchers do not interact with the respondents during the survey and thus cannot probe or oversee the data being collected, although research staff can be on site to respond to questions. Some of the methods often used to transmit and validate data such as XML or JavaScript can be lengthy and add to the amount of information that has to be passed between the client and the server. Often web forms are created as static (or pre-defined) forms that contain one long series of questions that require constant scrolling, which may interrupt the responder's thought process. Changes to static web forms can be difficult as they often require the interaction of a web developer with the researcher and, if the study has multiple questionnaires, tracking changes to these web forms can prove to be difficult. If the questionnaire has to be provided in multiple languages, creating the exact web form in each of the different languages can be challenging and time intensive. Additionally, creating a web-based questionnaire requires technical skills and facilities. Although the questionnaires are not vulnerable to virus infection, the computers at the remote locations are susceptible to viruses which can slow or incapacitate the ability to take the questionnaire. Finally, this type of data collection requires the remote computers to have a reliable connection to the internet, and there is always the threat of internet down time.

Although the evolution of numerous tools and methods has greatly improved over the years, the process of collecting accurate data can still be difficult. Most epidemiologists are regularly challenged with devising methods to accurately collect data for research, especially in situations where there are multiple study sites or involve offsite data collection. Often this is a multi-step process that involves creating a paper questionnaire, recording responses on the questionnaire, reproducing the questionnaire in some type of an electronic form through which the responses can be entered into a database, and finally creating variable labels and text from the questionnaire for statistical applications so that data can be further analyzed. This process is even further complicated if the questionnaire has to be provided in multiple languages. This evolutionary process introduces many stages where errors can be made that could lead to inaccurate results. There are now commercial packages that address many of these issues but some of these packages can be expensive or difficult to use and often require considerable customization in order to implement. We are offering a low cost alternative that addresses most of these limitations, especially for complex questionnaires that require tailoring to a specific study.

## Implementation

### Study description

The following case study is intended as an example and set of specific lessons learned though the use of a dynamic web-based system. This study in Starr County, Texas, funded through the National Institute for Occupational Safety and Health and The University of Texas Agricultural Center at Tyler, is a three-year cohort study of high school students to assess the student's work and health status. Located along the Texas-Mexico border, Starr County is economically disadvantaged, largely Hispanic, and the home to many farmworkers and their families. Regardless of work status, high school students (from three high schools in Year 1, one high school in Years 2 and 3) were asked to participate and to complete the web-based questionnaire in English or Spanish. The first two years of the questionnaire have been completed and because many of the students migrate with their families for farm work, data were collected in fall 2003 and fall 2004 to coincide with the off-migration season. The study seeks to describe work patterns and to identify risk factors for injuries in farmworker youth, and to compare farmworker adolescents with adolescents working non-farm jobs with respect to their work patterns, demographics, health status, health behaviors, and occupational injury.

In order to collect the data for this study, a complex research questionnaire was needed in both English and Spanish to be taken by respondents located at remote locations in rural South Texas approximately 260 miles from our university. The questionnaire for the first year was composed of seven parts containing 135 questions with 407 possible responses and incorporated skip patterns based on responses given by the student to specific questions (a similar questionnaire and process was developed for Year 2). For example, because the study was focused on farm work, after providing basic demographic data, the students were asked if they did farm work or non-farm work within the past nine months. If they responded "Yes", they were asked how many different employers they had, and a series of questions was asked about each of these employers. As the students entered their responses to questions in a language of their choice, the results were directly recorded into a database data table.

### Software details

Our main objectives for the data management needs of this study were to:

• create a system in which we could enter our questions and possible responses only once into a database table and then reuse this information for multiple purposes.

• use this system in future studies.

• create the tables needed to record the data entered through our questionnaire from this database table of questions and possible responses.

• produce a user friendly web-based questionnaire.

• provide data validation during the entry process.

• produce a coding manual that could be used as a reference document.

• generate basic statistics that could be viewed through the web during the entry phase of the questionnaire.

• produce the SAS and SPSS program files that could allow us to do more advanced statistics once the questionnaire was completed.

• have the ability to use a Microsoft Access database to access our questionnaire table and to access the data entered into the questionnaire. This database would allow us to use the features built into Microsoft Access such as forms, queries and reports to further work with the data.

**Figure 1:**
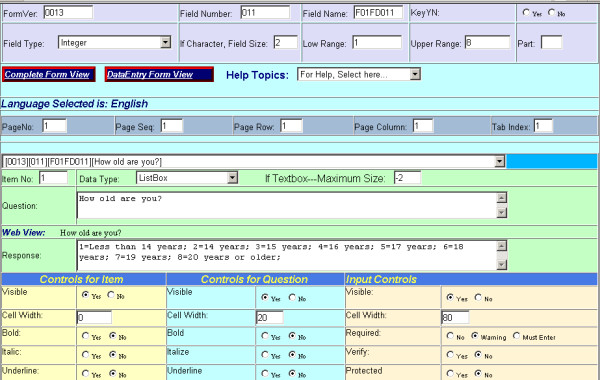
Questionnaire Database Management Web Page.

To accomplish our main objective of having a system in which the questions and possible responses could be used for multiple purposes, we created a database in our SQL server to store the data table of questionnaire information. The data table that stores the questions and responses also contains attribute fields that controls how the text is to be displayed, such as whether to turn on bolding or italics or to display certain information in specific colors. We then built a web form using the Microsoft ASP.Net programming language through which our questionnaire designers could enter and manage the questions and possible responses through a web browser. Figure 1 displays a snapshot of part of the web form used for managing the questionnaire database. Using ASP.Net code, we further enhanced our system by incorporating additional features that combined the questionnaire and data tables to produce web-based summary and crosstab reports, to reproduce the questionnaire as a web-based report and as a coding manual, and to create the coding files that could be used in commercial statistical packages to do more advance statistical analysis. To accomplish our Microsoft Access needs, we wrote a non-web-based application to generate an Access database that linked to our data stored in the SQL server and to produce Access data entry forms that closely matched the web-based questionnaires. Access provides resources (e.g., such as queries, forms, reports, and modules) that allowed us to locally manage the data and to generate various reports using the Access reporting features.

Although this system was developed for our study where data were entered from remote locations, this process of producing questionnaires from a database can be used for a number of research studies if internet connectivity is available and data are entered by the respondents. For example, this system could be useful for studies that enter data through web-based questionnaires at a central location.

The current software is limited to the Microsoft environment in which the researcher uses the Windows Operating system and the Microsoft Internet Information Services (IIS) and has the ASP.Net service enabled. To store the questions and responses, Microsoft SQL server is also required. Although the web-based application was configured to record data into an SQL server database, it can be configured to record the results into an Access database. However, there are restrictions of using the system in this configuration because of the limitations of using Access in a multi-user environment. As with any currently available commercial product, programming expertise is needed to customize this software to specific study needs and for data editing involving complex logic. A generic example questionnaire and the code used to produce this questionnaire are available for downloading [1].

## Results and discussion

Based on these first year data from over 2,500 respondents, it took an average of 17 minutes to complete the web-based questionnaire [18]. During the first year questionnaire, the most serious problem that occurred was loss of connectivity for some students during the middle of taking the questionnaire most likely because of the limited internet bandwidth to the school (affected 6% of the questionnaires). Each session had between 40 and 80 students taking the questionnaire at the same time. Unfortunately if connection was lost, students had to restart the questionnaire from the beginning if they had time before having to return to class. A back-up recovery process was implemented during the second year questionnaire which mostly eliminated the issue of students having to start over from the beginning.

We also encountered some other problems that were specific to some schools; however they could happen at other locations as well. At one school, prior to questionnaire administration, a major internet virus infected many of the computers in the lab, which limited the number of available computers to take the questionnaire. Ideally, research staff could have provided support to the schools to prepare the computer labs such as updating virus definitions and running virus scans a few days prior to questionnaire administration. At another of the schools, one of the labs lost internet connectivity and thus prevented it from being used for completing the questionnaire. The questionnaire is scheduled to be repeated again for the third and final year of follow-up and further adjustments will be made to enhance and improve on the system. Most of the problems encountered during the first year either were not encountered during the second year or were corrected in the ASP.net code used for the second year. For the third year questionnaire, we will be investigating and perhaps implementing the XML and XSL standards for distributing and displaying the questionnaire web forms and implementing the W3C committee proposed XForms standards for web-based forms.

In general, this questionnaire database approach [13] provided many advantages over a static web form approach. One advantage to this approach was that it allowed the questions to be entered only once through either a web-based or a Microsoft Access database form. This allowed non-programming staff to implement changes or enhancements controlled through either of these forms. The results were viewable immediately through a browser with a simple refresh. The database also made it much easier to convert the questions into the Spanish without risking changes to the overall questionnaire. Further, the questions and responses could easily be used to produce web-based reports through ASP.Net compiled code or ASP scripts or through the reporting tools built into Access. Examples of such reports include web-based data summaries, crosstab reports, and a code book report needed for documentation. It could also be used by custom applications to auto-generate the data tables needed to record the responses or to build control files such as those needed for other advanced statistical applications, thus eliminating the need to recode all the questions and responses for these applications.

The database approach also has disadvantages compared to the fixed or static web form approach. For example, the style or layout of the web-based form can be much more extensive with a statically generated web-based form because of the many features that commercial applications provide for these enhancements. In addition, a static approach to designing a web-based form provides more ease and flexibility in creating JavaScript with more elaborate error checking techniques than a dynamic approach. For example, it is much easier to create code to do cross field editing of responses because all the fields contained in the static web-based form are downloaded to the client at the time of connection. The paging technique used in this study only downloaded the fields needed to obtain responses for the questions contained on the page being displayed. Further, once a static web-based form has been created, it is easier to transfer and implement on other servers regardless of operating systems.

Although there are commercial products available that accomplish many of these tasks, for our project, we did not find any 'ready-made' product that we felt could accomplish all of our goals without significant customization. Further, as is often the case with competitive research grants, limited funding prevented us from purchasing a commercial product that would satisfy most of our requirements. Fortunately, we had the programming expertise to develop a web-based application that met the stated needs for our study.

Detailed instructions for installation and getting started with the data collection system have been provided through the download website. The following provides an overview of the process of implementing the system in studies of defined samples of respondents.

### Database and website requirements

To implement this dynamic web-based approach, a database must be created on a SQL server to hold the tables needed to store the questions, possible responses, and the data entered through the web-based questionnaire. A website also has to be created and configured in the IIS web server to house the ASP.Net application. Once the ASP.Net code for the application is copied to this site, a few statements in the database reference section of the code needs to be modified for the application to access the database. The code must then be compiled using Microsoft Visual Studios .Net.

### Entering and managing the question and response information

The process of entering a questionnaire will vary based on how the questionnaire is constructed in paper form. The system that is available to download is configured to allow for the entry and management of a typical questionnaire similar to the one used in this study. Once the application has been installed on a website, adding a new questionnaire through the web management page should be relatively straightforward. We will use our case study to describe what may be involved in this process. Our study was divided into several sections that comprised separate content areas to help guide respondents' thinking processes. Each section was assigned a unique number composed of a form number and a version number. Every data item in each section was assigned a sequential field number that could be used to uniquely identify the item within the entire questionnaire when combined with the section number. This combined identifier uniquely labeled the data record associated with each question in the table. This record contained the text for each question and other information that controlled how the question was to be displayed to the web. We used another data field in the table to store the possible responses for the respective question. The possible responses were coded in this field in the traditional style that is used by some statistical programs by using the coding value of each response followed by the text for the response. For example, for the question "How old are you?," the possible responses were coded in the database as "1 = Less than 14 years; 2 = 14 years; 3 = 15 years;" and so forth. Information was also maintained in the table to identify the calling statements for pre-programmed Java Scripts that were transmitted with the generated web forms in order to do entry edits. For example, this field could have information such as "rangecheck (1, 5)" which would signal that the "rangecheck" JavaScript should be used to check the respondents answer for a range between 1 and 5.

### Study specific customization requirements

Once the questions and responses have been entered through the web-based management page and are displayed in an acceptable manner, the questionnaire needs to be made available to the participants for access. We created a separate server application for this purpose. The reason that this application is independent of the web-based management component is because it needs to be accessible through a separate link that does not allow the participants access to the main system. Further, depending on the questionnaire, this application could be customized to better meet the needs for a specific study without having to make changes to the management system. For our study, as each part of the questionnaire was completed, the information was saved to the database into the respective section's data table and the next appropriate part of the questionnaire was displayed dependent upon the answers provided by the respondent. To enable this to happen, we had to incorporate some study specific custom code that could implement these skip patterns. The default application provided through the download is configured to read the questionnaire information from the database, format the question using the attribute fields and format the responses into the appropriate display types such as textboxes, radio lists, dropdown lists, or checkboxes. By changing the questions in the database, the application should work without modification for other similar questionnaires. The download example has all custom code removed but provides an example that demonstrates how to apply custom code to the system.

### System testing and logistics in current study

The process of testing the system was extensive. We created a number of paper forms of the questionnaires with pre-assigned responses. We made copies of these forms, distributed them to a number of researchers, and had each of them enter the responses independently into the system. We then compared the computer-stored results to the paper responses to ensure that all users entered the same data. We also had multiple users complete the questionnaire, each recording their responses on paper and then used the resulting computerized data to compare to what they recorded on paper for accuracy and completeness. We also printed out a coding book to be sure all questions had all possible responses that we had intended (hand verified).

### Security considerations

To ensure the security of the data as it passed through the network, we used the standard Secure Socket Layer protocol (SSL/HTTPS) for connecting to our questionnaire from the client. To incorporate this method, a server security certificate needs to be configured on the server. This certificate can be one that is created using the default utilities that are part of the Microsoft IIS server environment or one that is purchased from a commercial vendor.

### Displaying/editing the questionnaire

**Figure 2:**
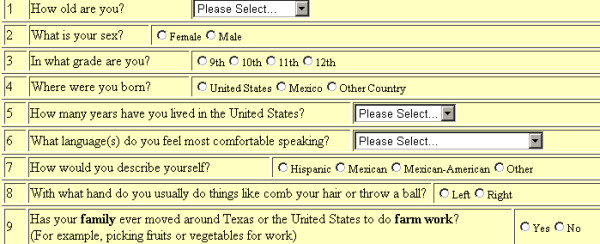
Web View of the Dynamically Generated Questionnaire Page.

The system was designed to allow the developer to configure the questionnaire so that it can be presented to the participants in multiple pages where only the number of questions that will fit on an 800 by 600 pixel viewing screen are displayed on each screen. Buttons are provided to allow the participant to move back or forward between the pages. This feature minimized the need for the user to scroll in order to respond to a question and thus possibly reduced the interruption to the users' thought process. The application also dynamically inserts the JavaScripts needed to edit the responses during the entry process when the page is displayed. Figure 2 displays a web view for one page of a questionnaire that was dynamically produced by the server application from the questionnaire data table.

For our study, measures were taken to make sure that the proper student responded to the questionnaire at the time of the visit to the high school. Prior to questionnaire administration, the study coordinator obtained a class roster from each school. These rosters were used to generate unique random numbers and a pre-assigned password for each student. Upon arrival at the computer laboratory, each student provided their name in exchange for a pre-generated card that contained the pre-assigned password and random number needed to access the questionnaire. They were instructed to enter this number with their pre-assigned password when prompted by the questionnaire web-based application. The random number and password were matched by the server to a master user's data table to ensure that the proper student was using the correct identifier. The random number would become their study identifier.

### Viewing the questionnaire results

**Figure 3:**
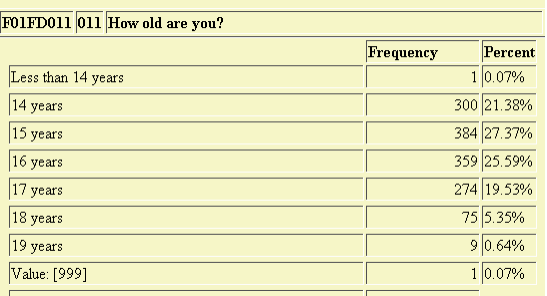
Web-based Dynamically Generated Statistical Summary.

**Figure 4:**
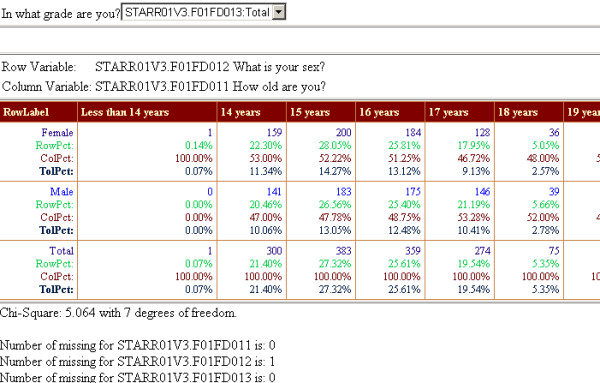
Web-based Select-Crosstab Report.

**Figure 5:**
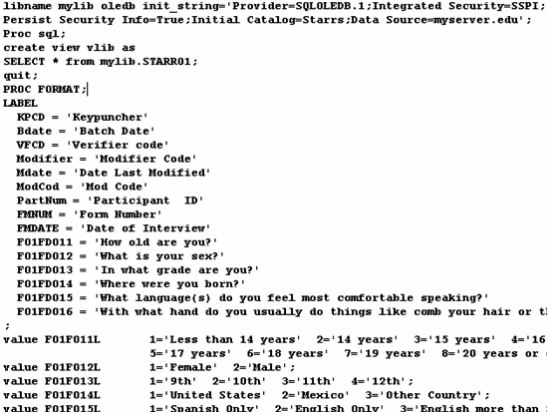
Input Templates from Questionnaire Database to Link to Statistical Packages.

**Figure 6:**
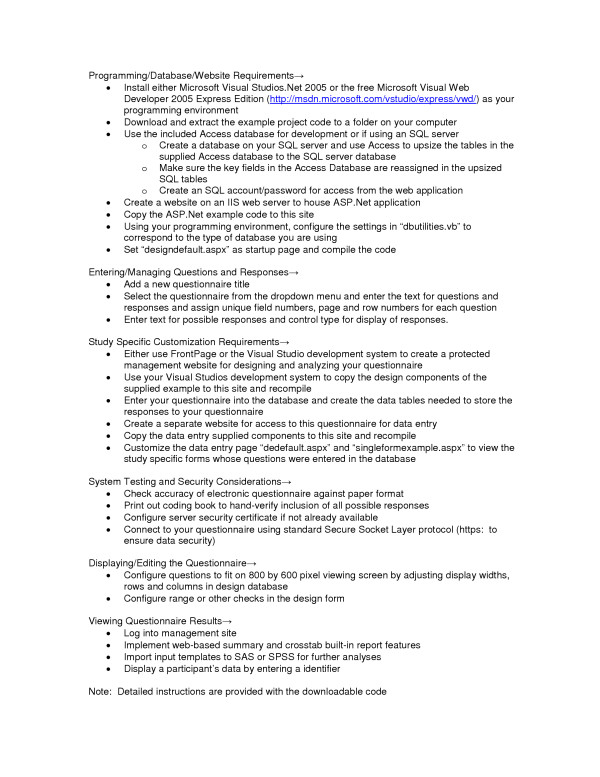
Flowchart of Steps Involved in our Web-based Data Collection Application.

As the students entered their responses to questions in a language of their choice, the results were directly recorded into a database data table. This information can be summarized at any time by the researcher by logging into to the management site and using the web-based summary and crosstab report feature. These reports are generated by combining both the questionnaire and the data tables to dynamically produce the desired web-based report (Figures 3 and 4). The application also produced input templates from the questionnaire database that could be used by SAS or SPSS to conduct further analyses of the results (Figure 5). A summary flowchart of the complete cycle of steps involved in our web-based data collection application is shown in Figure 6.

## Conclusion

In conclusion, web-based data entry using a dynamic approach proved to be a very efficient and effective data collection system. This data collection method expedited data processing and analysis and eliminated the need for cumbersome and expensive transfer and tracking of forms, data entry, and verification. With the noted enhancements, the third year of data collection should advance this methodology even further. This data collection method can be effectively applied to many other research areas using the techniques described in this paper.

## Availability and requirements

• Project Name: Web-based Data Collection, A Dynamic Approach.

• Project home page: 

• Operating System: Microsoft Windows 2000, 2003 or later with IIS web server enabled.

• Programming Language: Microsoft ASP.Net and JavaScript.

• Other requirements: Microsoft SQL server, Microsoft Access.

• Licenses: Microsoft licenses for the above applications.

• Restrictions for non-academics: The software is to be used for non-profit purposes only. Any enhancements are to be shared with the research community without cost and the authors should be provided with these enhancements so that they can be made available under the above website.

## Competing interests

The author(s) declare that they have no competing interests.

## Authors' contributions

CJC was the primary developer of the code and databases needed for this project. SPC was the principal investigator for the study and was responsible for the overall direction of the project and for the questionnaire design and implementation. DJDJ directed the data analysis for the study and reviewed the content of this document. EMS was the study coordinator and was responsible for the design of the questionnaire, the entry of the questions for the study, the careful testing of the web-based questionnaire produced and in the supervision of the numerous sessions in which the participants took the self-administered questionnaire. RW aided in the development and implementation of the questionnaire, and in providing analysis of the data obtained that were needed for this publication. SRC initiated the idea for this paper and was responsible for the literature searches and for reviewing and correcting the various drafts of this article. All authors read and approved the final manuscript.
